# A Comprehensive Model for Understanding Breast Cancer Screening Hesitancy: Integrating the Health Belief Model and the Confidence, Convenience, Complacency, Constraints, and Risk and Responsibility Calculations (5C) Model

**DOI:** 10.7759/cureus.71583

**Published:** 2024-10-16

**Authors:** Yang Liao, Suhaily Mohd Hairon, Najib Majdi Yaacob, Li Luo, Tengku Alina Tengku Ismail

**Affiliations:** 1 Department of Community Medicine, School of Medical Sciences, Universiti Sains Malaysia, Kubang Kerian, MYS; 2 Biostatistics and Research Methodology Unit, School of Medical Sciences, Universiti Sains Malaysia, Kubang Kerian, MYS; 3 Department of Oncology, Guihang Guiyang Hospital, Guiyang, CHN

**Keywords:** 5c model, barriers, breast cancer screening, health belief model, hesitancy

## Abstract

Breast cancer screening (BCS) is a critical preventive measure that can significantly reduce mortality rates. Despite its importance, screening hesitancy remains a global issue. This paper showcases the combination of the Health Belief Model (HBM) and the 5C Model and how it provides a more holistic understanding of BCS hesitancy. The first model, HBM, is a well-regarded tool that collects data based on individual beliefs such as perceived susceptibility, severity, benefits, barriers, cues to action, and self-efficacy. The second model, the 5C Model, stands for confidence, convenience, complacency, constraints, and risk and responsibility calculations. This model adds a layer of environmental considerations that HBM lacks. By combining these models, we can identify the key psychological, social, and structural barriers that contribute to BCS hesitancy. Furthermore, analysis of the literature suggests that enhancing trust in healthcare systems, increasing accessibility and affordability of screening, addressing cultural and social stigmas, and promoting a sense of collective responsibility can significantly improve screening participation rates, which are reflected in the models.

## Introduction and background

Introduction

The main preventative measure in reducing cancer mortality and mortality from other diseases is cancer screening [1]. Because of this, many countries incorporated screening programs into their medical guidelines. However, a significant number of people still refuse or are hesitant to get cancer screening. According to a report, all cancer-type cases will double within the next 50 years compared to 2020 [2]. This emphasizes the severity of cancer and shows how much of a barrier screening hesitancy poses in the battle against cancer.

One of the most common cancers among women is breast cancer. According to a 2024 report by the World Health Organization [3], in 2022 alone, breast cancer was the most prominent type of cancer among women in 157 out of 185 countries, resulting in 670,000 deaths worldwide. Breast cancer screening (BCS) hesitancy has been a contributing factor for this number. However, it affects many countries differently, ranging from a 1.7% breast screening rate in Bangladesh to 85.5% in the United Kingdom [4]. This disparity is also seen in the coverage rate of mammography in the different regions in Chile, ranging between 0.6% and 14.4% [4]. China, as one of the fastest developing countries, still suffers from a low BCS rate. From 2018 to 2019, for women aged 20 and above, the BCS rate was 22.3% [5].

In recent studies, it has been determined that individuals under certain demographics exhibit a higher level of hesitancy toward BCS. These individuals often have lower levels of education, lower income, and lower insurance coverage compared to their counterparts [6]. Other demographic factors, such as religious beliefs and ethnicity, can also affect individuals' acceptance of BCS [7]. Besides the demographic factors, there are psychological factors contributing to screening hesitancy. These include safety concerns, time requirements, and feelings of fear and embarrassment [6]. Therefore, to effectively increase BCS rates, it is important to understand how both demographic and psychological factors contribute to BCS hesitancy.

A high rate of BCS hesitancy can result in severe consequences, as it is a critical factor in determining the rate of early screening and detection for improving cancer treatment outcomes [1]. When screenings are delayed, breast cancer cases are diagnosed at more advanced stages, reducing the success chance of cancer treatment and increasing mortality rates [6]. Late detection also could limit treatment options, forcing patients to undergo more invasive procedures and higher medical costs [1]. Delayed BCS can also significantly impact patients' quality of life by prolonging recovery and emotional distress [6]. Addressing these delays is essential to improving treatment outcomes for women worldwide.

To further expand on BCS hesitancy, this study integrates two key models: the Health Belief Model (HBM) and the 5C Model. The HBM is a widely used framework that focuses on individuals' beliefs about health behaviors, such as perceived susceptibility to illness, severity of consequences, benefits of action, and barriers to taking action [8]. It also considers cues to action and self-efficacy as critical factors in influencing health-related decisions [8]. The 5C Model is comprised of confidence, convenience, complacency, constraints, and risk and responsibility calculation [9,10]. This adds an additional layer of environmental and systemic factors that the HBM does not account for. Analyzing these models creates a more comprehensive understanding of BCS hesitancy, unlike previous studies, which solely focused on individual beliefs or systemic factors. This approach highlights the importance of considering both personal and external influences on breast screening behavior.

Factors influencing BCS hesitancy

Psychological Barriers

Fear and anxiety: It is separated into two categories. First is the fear regarding the process of BCS, and second is the fear regarding the screening results. These two make up most of the psychological barriers [11]. In a study, more than half of the participants experienced intense fear, anxiety, and sadness when thinking about breast cancer [6]. In another study, Lebanese women reported feeling uncomfortable looking at their bodies during BCS, worrying about finding abnormalities in their bodies [12]. Another form of fear comes from physical concerns, such as potential discomfort, pain, stress, and radiation exposure during mammography and other screening procedures [13]. Limited understanding and misconceptions about BCS amplify these psychological barriers [8]. Besides the screening process, people are also afraid of the screening results, which creates extreme anxiety. This fear stems from anxiety about how to deal with positive cancer results or other serious health issues detected through screening [7]. Some individuals believe that a positive cancer diagnosis is equivalent to a death sentence, which may cause them to not undergo screening and continue living in ignorance [8,14].

Embarrassment: Feeling embarrassed and uncomfortable about examinations involving private areas. Embarrassment is a significant reason for not accessing screening facilities, even among individuals with easy access to healthcare and good education [15]. When considering BCS, women may encounter various psychological barriers, such as embarrassment related to physical exposure and the examination process, which is regarded as a significant factor influencing their decision to undergo screening [8]. Some Asian/Pacific Islander, African/Black, and American Native/Indigenous women feel embarrassed, ashamed, and shy with male doctors, which may increase their hesitancy level about mammography [16].

Privacy concerns: Issues about privacy and confidentiality, especially in smaller communities, can also affect participation rates in screening. A study in Canada showed that privacy concerns as a potential factor contribute to the reluctance of many people to be screened in the community or in public facilities [17].

Health self-assessment: When individuals think they are healthy or currently asymptomatic, they may think they do not need cancer screening because they are overconfident in self-diagnosis and underestimate their risk of developing breast cancer. Individuals think they are healthier than average, so they should leave the healthcare resources to those who need them more, which increases their chance of refusing screening [14]. This overconfidence could come from them believing they have a lower risk of developing breast cancer due to a lack of family history of cancer or because they exercise healthy habits [18]. Additionally, some believe that if they are over the age of 60, then their hormonal changes are within a "safe range" [19].

Knowledge and Awareness Barriers

Unawareness regarding BCS: Lacking detailed requirements regarding BCS procedures reduces BCS rate. Despite individuals claiming to be familiar with BCS, they may lack information or have misconceptions about its procedures, such as test intervals and age eligibility, which may reduce their participation rate [12]. Lack of knowledge and misunderstandings are significant barriers to BCS adherence. A study in the United Kingdom found that many women, including those who regularly undergo screening, knew little about the current National Health Service screening intervals and/or the age range for screening eligibility [20].

Misunderstanding risks: If there is a lack of accurate information regarding the risks of BCS, it could increase screening hesitancy. While BCS increases the detection rate of breast cancer, many individuals refuse BCS because they psychologically think screening guarantees a positive diagnosis [21]. Combined with their view of cancer being an incurable disease and fear that the treatment will cause extreme pain and other medical complications, they would choose to live in ignorance [20]. Furthermore, some doctors lack accurate information regarding the accuracy of screening, leading to either under-performing or over-performing screening procedures for patients based on their bias, and with overdiagnosis, there is a higher chance of false positives [22]. This will result in individuals believing that doctors are overestimating the accuracy of screening and would refuse screening for fear of overdiagnosis and false-positive results, which would put them in unnecessary treatment [21].

Social and Cultural Barriers

Distrust in the healthcare systems: Distrust could result from previous poor medical interactions, medical errors, information asymmetry, and skepticism toward medical institutions. These negative perceptions hinder individuals from proactively seeking BCS opportunities [23]. As noted by others, women feel that healthcare institutions are not considerate enough, such as causing them unnecessary pain and inconvenient schedules [19]. Additionally, lack of or difficulty in accessing medical resources may further contribute to a reduction in trust in the capacity of the healthcare system, thereby increasing the hesitancy level for BCS [24].

Sociocultural norms: Religious beliefs and cultural and social norms can lead to hesitancy to BCS [20]. In certain cultures, there could be barriers to spreading awareness and encouraging attitudes regarding BCS, as medical interventions are seen with fear or shame. This will result in individuals being reluctant to undergo screening [21]. Social norms such as traditional gender roles could also create a barrier for women in performing BCS, as some women might neglect their health due to family responsibilities [21].

Structural and Economic Barriers

Cost and accessibility: Financial cost is one of the main barriers to preventing individuals from accessing BCS services [20]. The Health Promotion Board in Singapore claims that cost barriers are one of the leading factors in low screening rates since the costs of BCS are paid by individuals' medical insurance [25]. Besides direct costs, other related costs such as transportation and lack of insurance also amplify these obstacles, especially for those in lower socioeconomic groups [26]. People in lower socioeconomic groups need to dedicate more of their time to meeting basic needs, making it difficult to allocate more money and time for BCS [27]. People of the average socioeconomic status also face accessibility issues, such as needing to take time off from work, arranging transportation and childcare, and managing follow-up screening appointments [27]. Furthermore, the operating hours of healthcare institutions often conflict with standard work schedules, making regular screenings more difficult [26].

Availability of BCS methods: The availability of different BCS methods, such as mammography, clinical breast exams, and self-examinations, is an important factor in the accessibility level of screening services. Mammography is one of the most effective early detection methods. The accessibility of mammography depends on the availability of mammography centers [8]. Urban areas typically have more screening centers, while rural and poorer areas often face a shortage of these facilities [18]. In China, disparities in screening rates between urban and rural areas are partly due to the uneven distribution of screening facilities [5]. The lack of screening facilities in rural areas increases individuals' travel time and costs for mammography, thus reducing participation rates [7]. Countries with limited investments in healthcare may lack digital mammography technology that provides more detailed images than traditional methods, lowering early detection accuracy for those without this technology [18]. Another problem in rural and low-income areas is the lack of public health outreach programs, which creates information gaps and awareness about BCS [13].

Accessibility factors: Another accessibility issue is related to quality facilities. Many women have difficulties finding conveniently located centers that offer services at convenient times. While more widely available, clinical breast exams still require trained healthcare professionals to perform high-quality examinations [8]. Countries with a shortage of trained healthcare professionals in breast cancer detection limit the availability of quality clinical exams [13]. In addition, self-examinations, which are low-cost and very accessible, depend on their effectiveness with the proper education on their process and awareness [6]. Unfortunately, there is inequality in accessing educational programs and awareness campaigns across different socioeconomic groups [7]. In low-income communities, the lack of access to accurate information may lead to underutilization or improper practices of self-examinations, further exacerbating disparities in screening participation rates [18].

## Review

The 5C model describing cancer screening hesitancy based on HBM

Theoretical Framework

HBM: The HBM was developed by social psychologists at the US Public Health Service in the early 1950s [25]. The purpose was to determine the factors why people did not participate in public health screening programs despite the availability of preventive measures [13]. Over time, HBM gained popularity and became a widely used theory for predicting individuals’ health behaviors, explaining their attitudes and decision-making regarding health-related issues [7]. HBM states that an individual’s behavior depends on six major factors: (1) perceived susceptibility, (2) perceived severity, (3) perceived benefits, (4) perceived barriers, (5) cues to action, and (6) self-efficacy [13]. In the context of BCS, a high level of perceived susceptibility and severity encourages an individual to undergo screening, while high levels of perceived barriers deter an individual from undergoing screening [26]. Even though a well-regarded model, the HBM does not account for the systemic and environmental factors in health behavior decision-making [25].

5C Model: The 5C Model was developed in 2018 by a German psychologist, Cornelia, to better understand vaccine hesitancy [9]. Due to its effectiveness, this model was then expanded and used in other areas of health behavior, such as cancer screening. As the name suggests, there are five major factors of the 5C Model to predict an individual’s decision-making regarding health-related issues [9]. The five determinants are confidence, convenience, complacency, constraints, and risk and responsibility calculation [9,10]. Confidence refers to an individual’s trust in the effectiveness and safety of the screening procedures, the trust in the reliability of the health services, and the competency of the health professionals and the policymakers [23]. Convenience refers to an individual's ability to get screening. It is determined by service availability, geography, and ease of communication such as language and health literacy [28]. Complacency reflects on an individual’s perceived risk and threat level of disease and the perceived necessity of screening [24]. Constraints include structural and non-structural barriers related to medical services [29]. According to the discussion [29], risk and responsibility calculation refers to an individual's perception of the risks associated with accessing health care procedures, as well as the level of responsibility one feels toward others, such as family or community. By incorporating the five determinants, the 5C Model helps in proving a more comprehensive framework for understanding hesitancy, thereby helping in developing more effective intervention strategies. Table 1 shows the difference between HBM and the 5C Model.

**Table 1:** Comparison and integration of the HBM and 5C Model HBM: Health Belief Model, 5C: confidence, convenience, complacency, constraints, and risk and responsibility calculations

Model components	HBM	5C Model
Perceived susceptibility	Recognition of personal risk of illness	Included
Perceived severity	Recognition of the seriousness of illness	Included
Perceived benefits	Recognition of the benefits of preventive or screening behaviors	Included
Perceived barriers	Recognition of obstacles to preventive or screening behaviors	Included
Cues to action	Internal or external signals that trigger health-related behaviors	Included
Self-efficacy	Belief in one's ability to perform specific behaviors	Included, expanded to include trust in the entire healthcare system and policymakers
Confidence	Not included	Trust in the safety and efficacy of screening, trust in the healthcare system, and policymakers
Convenience	Not included	Convenience in accessing screening services, including accessibility and willingness to pay
Complacency	Not included	Belief that the disease is not a threat and that screening is unnecessary
Constraints	Not included	Structural and non-structural barriers, such as political and socioeconomic obstacles, behavioral habits, social pressure
Risk and responsibility calculation	Not included	Evaluation of health risks of cancer treatment and screening, and the willingness to undergo screening to protect others (e.g., family members)

The HBM is a widely used framework for measuring psychological factors regarding BCS. However, one of the flaws that the HBM framework faces is the lack of environmental and systematic factors [25]. These factors are present in the 5C Model.

Analysis of using the 5C Model to measure BCS hesitancy

Confidence

In the HBM, confidence is described as self-efficacy. In the context of BCS, self-efficacy is defined as an individual’s belief in their ability to successfully schedule and complete the BCS [26]. In the 5C Model, confidence encompasses the self-efficacy from HBM and includes the factor of trust in the healthcare system and the healthcare policymakers [28]. For example, a woman believes in her ability to schedule and go through the process of BCS, but if she lacks confidence in the local medical facilities, believing that the medical equipment is outdated or the medical professionals are not professional enough, she will still hesitate to undergo BCS [14]. Lack of confidence in the government’s health policies, such as poor regulation on safeguarding personal information, will also create hesitation in getting BCS [8]. These perceptions are also shaped by social influences and past experiences with the healthcare system. For example, a previous negative experience with medical procedures or knowing others who have had such experiences might further diminish confidence in pursuing screening [18]. Therefore, building a robust and trustworthy healthcare infrastructure, alongside transparent and empathetic communication, is essential to bolster confidence and encourage participation in cancer screening [13].

Convenience

Inconvenience and time consumption are factors of the HBM perceived barriers dimension. These factors are also mentioned in 5C under convenience. Convenience is measured by looking at two parts, accessibility and feasibility. Accessibility factors include ease of scheduling, clarity of information, and information availability [18]. When information regarding the location, cost, and procedure of screening is easily available and easy to understand, women are more likely to perceive the screening as convenient [28]. User-friendly online platforms and informative brochures can significantly increase accessibility [26]. Feasibility is an individual’s ability to attend appointments and screenings or adhere to prescribed regimens without causing significant disruption to their daily responsibilities [14]. Feasibility could be improved by implementing flexible screening schedules, such as offering weekend or evening slots, reducing the risk of conflicting with employers for time off to participate in screening [13].

Complacency

Perceived severity and perceived susceptibility refer to an individual's perception of the seriousness and the risk of contracting a disease [13]. Severity indicates the potential impact of the disease, such as disability, long-term complications, or even death [27]. Susceptibility refers to the likelihood or predisposition of an individual to develop a particular disease [25]. These two factors correspond to "complacency" in the 5C Model. Studies have shown when a woman believes her risk of developing breast cancer is low or that the impact of cancer on her life is low, she is less likely to feel the need to undergo screening, while if she discovers her family has a history of breast cancer, her perceived risk will increase, reducing her complacency [30].

“Cue to actions,” when used effectively, can significantly influence the perceived severity and susceptibility [26]. Examples of “cue to actions” are media coverage that reports on increasing rates of breast cancer and survivor stories and educational programs that provide detailed information about breast cancer risk factors, symptoms, and benefits of early detection [8]. These can play a crucial role in addressing complacency and heightening awareness, prompting individuals to reassess their own risk, and pushing them to take a more proactive stance on BCS [28]. This can further be reinforced by social influences, such as recommendations from friends and family [16]. By understanding and addressing the factors contributing to complacency, healthcare providers can design more effective communication tools to spread awareness and encourage proactive healthy behaviors [18].

Constraints

The perceived barriers in the HBM overlap with the constraints factor in the 5C Model. However, the constraints in the 5C Model are classified into structural and psychological barriers [28]. Structural barriers contain political, social, and economic challenges [19]. An example of a political barrier is insufficient government investment in public health, which leads to limited screening services [7]. An indirect political barrier is a lack of infrastructure in rural areas, resulting in unequal distribution of medical resources and hindering individuals' access to screening services [18]. An example of social barriers could be observed in some traditional conservative societies. Women discussing or undergoing BCS may be seen as committing a taboo or doing a shameful act. If private body parts are seen by outsiders, they could be labeled as “impure,” thus, to avoid the social stigma many refuse to participate in BCS [22]. Another constraint is economic barriers, such as the cost of BCS, which creates a significant barrier for low-income individuals, even with partial subsidies [8]. In addition, the lack of healthcare insurance can increase this problem, as the cost of routine BCS adds up quickly [18]. For psychological barriers, fear is the primary factor that significantly affects the participation rate of BCS [20]. Specifically, fear of pain during mammograms, fear of radiation exposure, and fear of misdiagnosis all contribute to the reluctance regarding BCS [16]. The combination of these factors hinders timely and potentially life-saving diagnoses, collectively undermining the effectiveness of BCS programs.

Risk and Responsibility Calculation

The HBM includes two factors in risk calculations. They are perceived susceptibility and perceived severity. In BCS, perceived susceptibility relates to individuals’ perception of the potential harms, complications, and impact of breast cancer on their quality of life [30]. Perceived susceptibility relates to individuals’ perception of their own risk of developing breast cancer [13]. These factors align with the "risk and responsibility calculation" in the 5C Model. The 5C Model contains similar factors, such as the perceived risk of screening and the perceived benefits of early screening.

The 5C Model identifies three types of responsibility: personal responsibility, interpersonal responsibility, and societal responsibility [29]. Personal responsibility pertains to the extent an individual believes their actions impact their long-term health [9]. Interpersonal responsibility involves the degree to which an individual feels responsible for the health of those close to them, such as friends or family [29]. Societal responsibility refers to how much an individual feels accountable for the health of their community or larger society, including people outside their immediate circle [23]. The "collective responsibilities," such as interpersonal and societal responsibilities, are unique to the 5C Model and are not addressed in the HBM. These responsibilities are crucial because, in many cultures, women often serve as the primary caregivers within families. A mother who undergoes regular BCS sets a health-conscious example for her family, encouraging them to prioritize their health [13]. Early detection and treatment can also prevent the need for extensive medical care, reducing the burden on her family and allowing her to continue caring for her children and elderly relatives [26]. Additionally, when women actively participate in BCS and share their experiences, they raise health awareness within their community [7]. By encouraging others to get screened, they help improve the overall health of the community. This collective behavior not only enhances individual health outcomes but also strengthens the community's resilience against health crises [13]. Applying these models to BCS allows for a detailed understanding of the multifaceted factors that influence a woman's decision to participate in screening programs. Table 2 clearly displays each component of the 5C Model along with its measurement strategies and example questions, helping to comprehensively understand and assess various aspects of BCS hesitancy.

**Table 2:** Integration of the 5C Model for measuring breast cancer screening hesitancy 5C: confidence, convenience, complacency, constraints, and risk and responsibility calculations, BCS: breast cancer screening

5C Model component	Description	Measurement strategies	Example questions
Confidence	Trust in the safety and efficacy of BCS, the medical professionals conducting the screening, and the overall healthcare system	Design surveys to assess women's trust in the healthcare system, confidence in the abilities of healthcare providers, and confidence in the accuracy and safety of screening procedures. Include questions about past medical service experiences and their impact on current trust levels	1. The information I receive from the screening program (mammography) is reliable? 2. Breast ultrasounds provided by the government in my community are beneficial?
Convenience	Accessibility of screening services, including location, affordability, and availability of flexible scheduling	Assess logistical barriers such as travel distance to screening centers and availability of appointments outside typical working hours. Evaluate the clarity and accessibility of information about screening services	1. Do you believe BCS is accessible when you need it? 2. Is it easy for me to schedule a mammography?
Complacency	Perception of the threat of breast cancer and the perceived necessity of screening	Measure perceived severity and susceptibility to breast cancer. Explore the influence of family history, personal health beliefs, and media consumption on complacency	1. Is getting a BCS for preventing a disease that you are unlikely to get? 2. Do you think regular BCSs are important for you?
Constraints	Structural and psychological barriers, such as political and sociocultural obstacles, and psychological distress	Identify specific psychological barriers (e.g., fear of pain or embarrassment) and structural barriers (e.g., lack of healthcare resources in rural areas). Assess sociocultural factors that might prevent women from participating in screening	1. May getting a mammography be very painful? 2. Do you think certain ethnic groups in your community/area face difficulties in getting a BCS?
Risk and responsibility calculation	Weighing the health risks of screening and the responsibility toward oneself and others (e.g., family)	Evaluate how women assess the risks and benefits of screening. Include the influence of family responsibilities and collective health benefits	1. Do you feel a responsibility to undergo screening to ensure you can care for your family? 2. Does my participation in BCS encourage participation among others in the community?

## Conclusions

This review integrates the HBM and the 5C Model to provide a more comprehensive framework for understanding BCS hesitancy. One key advantage of this approach is its ability to capture both individual psychological factors, emphasized by the HBM, and external systemic influences, which are central to the 5C Model. This approach offers a more holistic understanding of the multifaceted barriers to BCS, providing a more solid foundation for developing targeted healthcare programs. However, this review is limited in its effectiveness due to the lack of direct empirical evidence in comparison of the two models’ effectiveness in various contexts. Most of the current literature focuses on a qualitative synthesis, and the 5C Model's relatively new application to BCS limits the availability of research specifically in this domain.

Future studies should empirically test this integrated framework across diverse populations to validate its utility and adaptability. This creates a better understanding of how to tailor the model to a specific cultural, socioeconomic, and geographical barrier to BCS. The integrated model can guide public health policies in creating more effective educational campaigns, improving access to screening services, and developing culturally sensitive interventions to address hesitancy by providing more informed healthcare professionals and policymakers, ultimately increasing screening rates and improving overall breast cancer health outcomes.
